# The nitrite reductase activity of xanthine oxidoreductase sustains cardiovascular health as mice age

**DOI:** 10.1016/j.redox.2025.103923

**Published:** 2025-11-07

**Authors:** Nicki Dyson, Rayomand S. Khambata, Tipparat Parakaw, Gianmichele Massimo, Ngan HH. Khuat, Annika A. Noor, Lorna C. Gee, Ivy Lim, Umme Siddique, Andrew J. Sullivan, Jonathan W. Ho, Krishnaraj Rathod, Michael R. Barnes, Claudia P. Cabrera, Amrita Ahluwalia

**Affiliations:** a Barts & The London Faculty of Medicine & Dentistry, Queen Mary University of London, Charterhouse Square, London, EC1M 6BQ, United Kingdom; b Department of Pharmacology, Faculty of Dentistry, Mahidol University, Thailand

**Keywords:** Hypertension, Inflammation, Nitrate, Nitric oxide, Xanthine oxidoreductase

## Abstract

**Background:**

Xanthine oxidoreductase (XOR) is a multi-functional enzyme that metabolises purines generating uric acid and generates reactive oxygen species. Both functions have been implicated in the pathogenesis of cardiovascular disease. More recently, a third function of XOR as a nitrite reductase has been identified. This nitrite reductase activity has been proposed to play a key role in the benefits of targeting the non-canonical pathway for nitric oxide (NO) generation in the cardiovascular disease setting; an effect specifically attributed to XOR-dependent recovery of NO levels. However, whether XOR derived NO plays any role in maintaining cardiovascular homeostasis in health is unknown. To explore this, we used global and hepatocyte-specific *Xdh*-deleted mice to assess cardiovascular homeostasis.

**Methods:**

*Xdh*^+/+^ and *Xdh*^+/−^, *Xdh*^*fl/fl*^*and Xdh*^*fl/fl*^*AlbCre*^*+/−*^ (HXOR KO) mice littermates, matched for sex and age, were used for *in vivo* cardiovascular phenotyping: blood pressure, cardiac function, endothelial reactivity, and leukocyte trafficking. Tissues were used for biochemical measurements of nitrate, nitrite, and measurement of markers of NO downstream signalling.

**Results:**

*Xdh^+/−^* and HXOR KO mice expressed significantly attenuated liver and plasma nitrite reductase activity and platelet cGMP levels versus littermate controls. As mice aged *Xdh-deficient* mice developed increasing systolic blood pressure, left ventricular remodelling, endothelial dysfunction and increased leukocyte activation versus their age and sex matched littermate controls. Endothelial dysfunction was reflected by increased endothelial adhesion molecule expression (P-selectin), increased ischaemia-induced vasoconstriction, during vessel occlusion, and an impaired flow-mediated dilation response of the iliac artery *in vivo*. Furthermore, the absence of XOR eliminates the benefits of dietary inorganic nitrate treatment.

**Conclusions:**

In summary, XOR derived NO is critical for maintaining, during ageing, vascular homeostasis under physiological conditions and is key in mediating the benefits of dietary nitrate regimes in cardiovascular pathology.

## Graphical abstract

**Figure:**
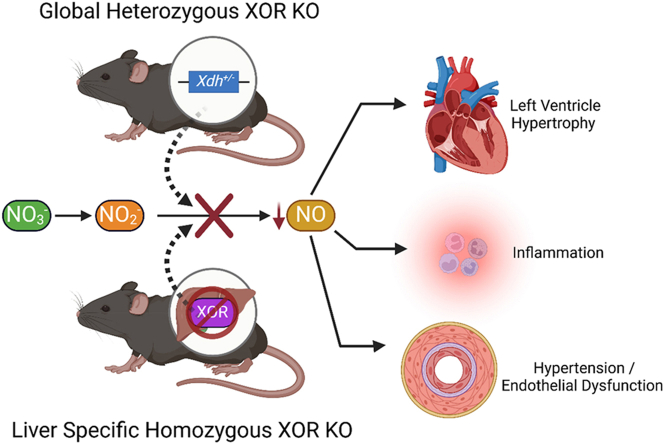

## 1 Introduction

Xanthine oxidoreductase (XOR), encoded by the *XDH* gene in humans and *Xdh* in mice, is a molybdoflavin enzyme expressed widely at low level but with high expression in the liver [1]. Whilst XOR is localised to the cytoplasm of diverse cell types of the cardiovascular system [2,3], it is also found on the extracellular surface. This cell surface expression is derived predominantly from the liver, where hepatocyte-derived XOR is secreted into the circulation from where it then readily binds to glycosaminoglycans (GAGs) on proteoglycans on the surface of endothelial cells [2], and probably red blood cells (RBCs) [3]. XOR catalyses the oxidation of hypoxanthine to xanthine and xanthine to uric acid (UA). These reactions generate a flow of electrons from the molybdenum site to the flavin adenine dinucleotide (FAD) site which, in the presence of oxygen, results in the production of reactive oxygen species (ROS), namely superoxide (O_2_^−^) and hydrogen peroxide (H_2_O_2_). Clinical studies show elevation of XOR activity and expression in patients with hypertension and coronary artery disease [[4], [5], [6]]. In addition, numerous pre-clinical *in vivo* studies suggest that xanthine oxidase derived ROS are, in part, responsible for the leukocyte recruitment observed in ischaemia reperfusion injury [7]. These data have been taken to collectively imply that elevated XOR activity and/or expression is pathogenic, due likely to elevated ROS and/or UA generation [8].

A hallmark of cardiovascular disease is endothelial dysfunction which is synonymous with a lack of bioavailable nitric oxide (NO), in part due to scavenging by ROS [9,10]. Whilst XOR is well recognised as a ROS generator, evidence indicates that a third function of the enzyme is its capacity to reduce nitrite to NO [[11], [12], [13], [14]]. This activity has been implicated as a key second step in non-canonical (l-arginine/NO synthase independent) NO generation [15,16]. Briefly, inorganic nitrate, found in green leafy vegetables, enters the circulation, concentrating within the salivary glands. Nitrate-rich saliva is excreted into the oral cavity where the nitrate is chemically reduced to nitrite by commensal bacteria expressing nitrate reductases [17,18]. This nitrite-rich saliva once swallowed enters the circulation [19] where it is reduced to NO. Several distinct nitrite reductases have been identified, however there is growing support for the importance of XOR as a nitrite reductase [20]. The positive effects of NO derived from the non-canonical pathway have been demonstrated in pre-clinical experimental models and in humans in both health and disease. However, whilst XOR has been strongly implicated as an effective NO generator in disease settings, what role it might play as a nitrite reductase in cardiovascular health is largely unknown. Thus, in this study we investigated the role of XOR-dependent nitrite reductase activity in cardiovascular homeostasis utilising *Xdh* transgenic mice. Additionally, to overcome the lethality of homozygous *Xdh* transgenic mice, the limitations of pharmacological XOR inhibitors and to investigate a proposed primary source of systemic XOR, we generated a unique hepatocyte (Alb-Cre) specific XOR-deletion mouse.

## 2 Methods

For expanded methods see online supplement**.**

### 2.1 Animal studies

All experiments were conducted according to the Animals (Scientific Procedures) Act 1986, United Kingdom, and approved by the UK Home Office. Unless stated otherwise, terminal procedures were performed under anaesthesia induced with 5 % isoflurane in 1.5 L/min O_2_ and maintained with 2.5–3.0 % isoflurane. Blood was collected via cardiac puncture using a 23 G needle attached to a 1 ml syringe pre-loaded with 70 μl of 3.8 % (w/v) sodium citrate (Sigma-Aldrich) to prevent coagulation. Secondary confirmation of death was achieved via cervical dislocation.

### 2.2 Generation of *Xdh* global and *Xdh*–hepatocyte specific (HXOR KO) knockout mice

**Fig. 1:**
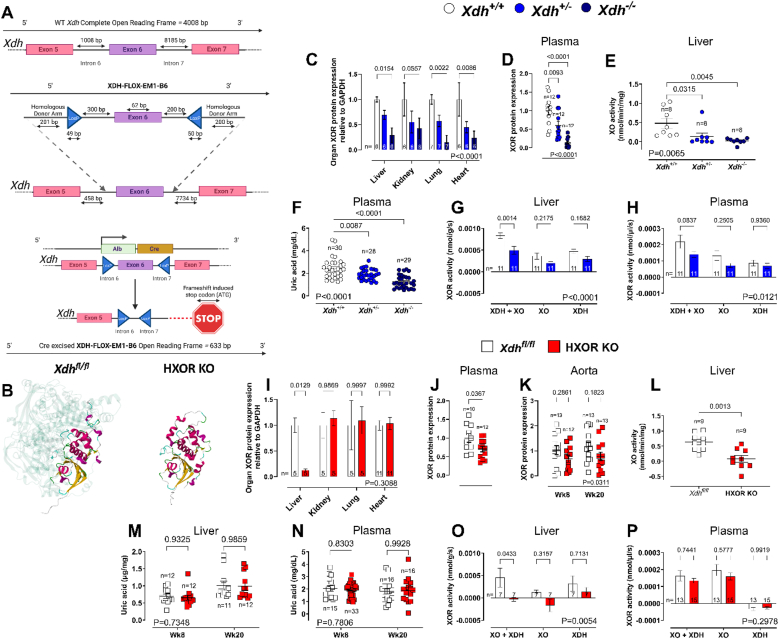
**Confirmation of genetic deletion of *Xdh*. (A)** Schematic of WT *Xdh* gene and insertion of XDH-FLOX-EM1-B6 via CRISPR/Cas9. The expression of the loxP regions in mice containing XDH-FLOX-EM1-B6 (*Xdh*^*fl/fl*^) results in Cre mediated excision of exon 6 in mice expression Cre recombinase downstream of the albumin promoter (HXOR KO). **(B)** Complete *Xdh* open reading frame in *Xdh*^*fl/fl*^ and HXOR KO mice with 1–633 bp highlighted and integrated into remaining *Xdh* open reading frame protein and formation of an XOR monomer. Western blot quantification of XOR protein expression across **(C)** various tissues and **(D)** plasma in the 4-week-old global *Xdh* deleted mice and their wild type littermate controls. **(E)** XO activity as assessed by amplex red H_2_O_2_ production and **(F)** plasma uric acid concentration and total XOR activity (XDH and XO isoform activity) in the **(G)** liver and **(H)** plasma of samples collected from 4-week-old *Xdh*^*+/+*^, *Xdh*^+/−^ and *Xdh*^*−/−*^ mice where available. Western blot quantification of XOR protein expression across **(I)** various tissues in 8-week-old mice, **(J)** plasma in the 20-week-old mice, and **(K)** the aortas of 8- and 20-week-old HXOR KO mice versus the *Xdh*^*fl/fl*^ wild type littermate controls. **(L)** liver homogenate XO activity assessed by amplex red H_2_O_2_ production in 20-week-old HXOR KO mice versus the *Xdh*^*fl/fl*^*.***(M)** Liver and **(N)** plasma uric acid concentrations in 8 and 20-week-olld HXOR KO and *Xdh*^*fl/fl*^ mice and total XOR activity (XDH and XO isoform activity) in the **(O)** liver and **(P)** plasma of 20-week-old *Xdh*^*fl/fl*^ and HXOR KO littermate mice. Data are shown as mean ± SEM of n mice (shown on individual graphs). Statistical significance for genotype was determined using two-way ANOVA (shown in bottom right **(C**, **G-I**, **K**, **M-P)** followed by Dunnett's *post hoc* test **(C and I)** or Sidak's multiple *post hoc* test **(G-H, K, M-P),** by One-way ANOVA **(D**–**F)** followed by Dunnett's multiple *post hoc* test highlighting significant difference from *Xdh*^+/+^ or by unpaired Student's t-test **(J and L).**

*Xdh* knockout mice (*Xdh* ^*−/−*^) originally generated by Finkel and colleagues [21], were bred in-house. Embryos were purchased and rederived at MRC Harwell in C57BL/6J mice and *Xdh^+/−^* breeding trios established. A novel C57BL/6J *Xdh* floxed mouse was generated via CRISPR/Cas9 genome editing (MRC Harwell) and then crossed with C57BL/6J Albumin (Alb) Cre mice (purchased from The Jackson Laboratory) to generate hepatocyte specific XOR knockout (HXOR) mice in-house (Fig. 1A and B). Both male and female littermate *Xdh*^+/+^, *Xdh*^+/−,^ *Xdh^−/−^* and *Xdh*^*fl/fl*^ control/HXOR KO mice were used in all studies.

### 2.3 Dietary nitrate protocol

Where required, mice (6 weeks old) were randomly assigned to receive drinking water supplemented with potassium nitrate (KNO_3_, 15 mmol/L) or potassium chloride (KCl, 15 mmol/L, control) for 2 weeks using previously extensively validated protocols [[22], [23], [24], [25]] and the dose selected based upon findings demonstrating a rise in circulating nitrite levels similar to that achieved in human studies [22]. For intravital microscopy studies, mice (3 weeks old) were assigned to the above protocol for 1 week only.

### 2.4 Tail cuff blood pressure measurement

Tail cuff blood pressure (BP) data was collected using the non-invasive CODA mouse BP system (Kent Scientific Corporation, USA) and the acquisition conducted blind to genotype.

### 2.5 Echocardiography

Transthoracic echocardiography was performed using a Vevo 3100 imaging system with a MX550D, 40 MHz transducer (FujiFilm VisualSonics Inc., Netherlands). Image analysis was conducted offline and blind to genotype [23].

### 2.6 Flow mediated dilation (FMD)

FMD of the arteria iliaca externa was visualized using a Vevo 3100 imaging system with a MX550D, 40 MHz transducer (FujiFilm VisualSonics Inc., Netherlands). An O-cuff (Kent Scientific Corporation, USA) was placed above the left hind leg knee and inflated to 300 mmHg at T = 0 min to occlude blood flow through the left arteria iliaca externa. At T = 5 min the cuff was released and arteria iliaca externa was visualized until T = 10 min [26]. Image analysis was conducted offline and blind to genotype.

### 2.7 Intravital microscopy to determine leukocyte recruitment

4-week-old mice were anaesthetised, the mesentery exposed and superfused with bicarbonate-buffered solution (BBS) and leukocyte rolling and adhesion counted as described previously [22].

### 2.8 Flow cytometry

Blood was collected and flow cytometry conducted to determine leukocyte subtypes, activation markers and XOR expression. Additionally, bone marrow was isolated and CXCR2 expression determined.

### 2.9 Measurement of nitrate and nitrite levels

Plasma and tissue nitrite (NO_2_^−^) and nitrate (NO_3_^−^) levels (collectively termed NO_x_) were measured using ozone-based chemiluminescence as described previously [22].

### 2.10 Liver homogenate nitrite reductase activity

The nitrite reductase activity of liver supernatants was determined using gas-phase chemiluminescence [27] at pH 7.4 (representing physiological conditions) and pH 6.8 (blood acidosis – conditions that also favour nitrite reductase pathways).

### 2.11 Immunohistochemistry

Liver and heart samples were collected from paraformaldehyde infused mice, sections prepared and stained with H&E, anti-CD62P, anti-CD45 and picrosirius red. Wheat germ agglutinin conjugated to Alexaflour 647 fluorescent staining was conducted in order to determine cardiac myocyte area [23].

### 2.12 Western blotting

Equal amounts of protein homogenates of liver or aorta were subjected to Western blotting to determine XOR, phosphorylated (p)-eNOS, total eNOS, VASP and pVASP expression levels.

### 2.13 Quantitative reverse transcriptase-polymerase chain reaction (qRT-PCR)

Liver and heart samples were subjected to qPCR and analysed using the comparative threshold cycle (Ct) method (2−ΔΔCt). See Table S1 for a full list of primers.

### 2.14 RNA sequencing analysis

RNA was extracted from cardiac homogenates of global *Xdh*^*+/+*^ and *Xdh^+/−^* mice and subjected to bulk RNA sequence analysis.

### 2.15 Liver XO activity

XO activity of liver homogenates was determined using a commercially available kit, according to manufacturer guidelines (xanthine oxidase assay kit, Abcam, UK).

### 2.16 Pterin-based fluorometric assay of XDH/XO activity

To determine the relative proportions of XDH versus XO activity in tissues and plasma we used a modified pterin-based fluorometric assay protocol as described previously [28].

### 2.17 Plasma and urine biochemical analysis

Whole blood was obtained via cardiac puncture into a syringe containing 3.8 % sodium citrate centrifuged at 14,000 rcf for 5 min at 4 °C and plasma collected. Plasma and spot urine samples were collected at the time of sacrifice, snap frozen and stored at −80 °C. Plasma and urine samples were analysed by MRC Harwell Pathology using the AU680 Analyser (Beckman Coulter).

### 2.18 Sample size estimation

Preliminary exploratory studies were conducted to assess global KO anion levels and/or BP in age- and sex-matched littermate mice. For these experiments formal sample size estimates were not conducted. For all echocardiography studies, power calculations were based on the sample size required to detect a significant difference in left ventricular (LV) posterior wall thickness. In previous studies, we demonstrated that in mice treated with 2 mg/kg/day Angiotensin II, LV wall thickness in KCl placebo control was 1.3 ± 0.4 mm (mean ± SD), compared to 0.81 ± 0.2 mm in mice treated with KNO_3_^26^. Using these values, with α = 0.05 and a power of 95 %, the required sample size was calculated to be n = 12 animals per group. To account for potential technical failures or phenotype-related loss of animals, the sample size was increased to n = 15 per group for both mouse models. Tail-cuff BP measurements were also performed in *Xdh*^*fl/fl*^ and HXOR KO mice that underwent echocardiography (n = 15). For qPCR, initial assessments of liver TNFα mRNA expression in 4-week-old *Xdh*^*−/−*^ mice yielded a relative expression of 1.0 ± 0.66 (mean ± SD), while in *Xdh^+/−^* mice, relative expression was 2.0 ± 0.77 (effect size 1.38). Using these values and a power of 95 %, a sample size of n = 15 mice per group was determined to be sufficient for statistical significance. However, for all qPCR and biochemical analyses, all available samples were utilised, on occasion exceeding n = 15. All groups compared were matched for sex, age, and littermate status.

### 2.19 Statistical and data analysis

Data are expressed as mean ± SEM of n mice. Statistical significance was determined using unpaired *t*-test, one-way ANOVA followed by Dunnett's post hoc *t*-test or two-way ANOVA with Sidak's post-test as required. A P value < 0.05 was considered significant. Gaussian distribution and homogeneity of variances of datasets were determined by the Shapiro-Wilk test and Bartlett tests, respectively. For all experiments n values were equal by design however where unequal n values within experiments are shown this relates to technical failure or from identification of outliers using the ROUT [29] exclusion test. Where any exclusions have occurred, this has been stated explicitly in the figure legends. All analysis was conducted using GraphPad Prism 10.1.2.

## 3 Results

### 3.1 Biochemical and molecular characterisation of global and liver specific *Xdh* transgenic mice

As anticipated, typical numbers of offspring and growth rates were observed in the global *Xdh*^*+/+*^ *and Xdh^+/−^* mice, while the *Xdh*^−/−^ mice had stunted growth with lethality by ∼4 weeks of age (Fig. S1A and S1B) [21]. Consequently, samples from *Xdh*^−/−^ mice were unavailable for non-juvenile (>4-weeks-old) *in vivo* studies. Whilst weight was similar between genotypes at 4 and 12 weeks of age, at 20 weeks *Xdh^+/−^* mice were modestly heavier than their *Xdh*^*+/+*^ littermates (Fig. S1A). Although when expressed as a change from wild type no statistical differences between *Xdh^+/−^* and *Xdh*^*+/+*^ were evident. In contrast to the *Xdh*^−/−^ mice, there were no survival issues observed in the HXOR KO mice compared to control *Xdh*^*fl/fl*^ littermates (Fig. S1C).

Global *Xdh* excision in *Xdh*^−/−^ mice was confirmed by assessment of mRNA (Fig. S1D) and protein expression (Fig. 1C and D and Figure S1F, G) in several tissues and plasma. In contrast, in HXOR KO mice at 8 weeks of age, *Xdh* mRNA (Fig. S1E) and XOR protein expression (Fig. 1I and Figure S1H) were profoundly reduced in the liver only, whilst expression in other solid organs remained unaltered. HXOR KO mice had a significant but modest reduction of plasma XOR expression (Fig. 1J and S1I) accompanied by a corresponding decrease in XOR expression in aorta homogenates, at 8 and 20 weeks (Fig. 1K and S1J), confirming that hepatocyte XOR contributes to circulating XOR levels and distant organ binding.

Purine-driven XO activity (in terms of ROS generation-measured as H_2_O_2_) was significantly reduced in liver homogenates of *Xdh^+/−^* mice versus *Xdh*^*+/+*^ mice and entirely absent in *Xdh*^*−/−*^ mice, (Fig. 1E). Similarly, purine-driven XO activity was significantly reduced in liver homogenates of HXOR KO mice in comparison to the *Xdh*^*fl/fl*^ mice (Fig. 1L), despite no difference in hepatic basal production of O_2_^•-^ or H_2_O_2_(Fig. S1K). Interestingly, whilst an allele dependent reduction in plasma UA levels was evident in the global deletion mice (Fig. 1F) no such effect was observed in plasma or liver homogenates of HXOR KO mice (Fig. 1L). XDH and XO activity proportions of XOR were both decreased similarly in the global deletion mice in liver (Fig. 1G) and plasma (Fig. 1H). In contrast, although a complete ablation of XDH and XO activity was evident in liver samples of HXOR KO mice (Fig. 1O), only a modest reduction in plasma activity was evident, which did not reach statistical significance (Fig. 1P).

No overt changes in general circulating biochemical parameters were evident in 4-week-old global KO mice and 20-week old HXOR KO mice, except for urea levels which were reduced in the global *Xdh^+/−^*mice only (Table S2), with no difference in the HXOR KO mice aligning with previous observations [21,30]. Importantly, neither *Xdh^+/−^* or HXOR KO mice expressed differences in markers of liver damage, including ALP, AST and ALT, compared to their wild type littermate controls (Table S2).

### 3.2 *Xdh* deletion alters nitrite reductase activity and influences nitrite and nitrate dynamics

**Fig. 2:**
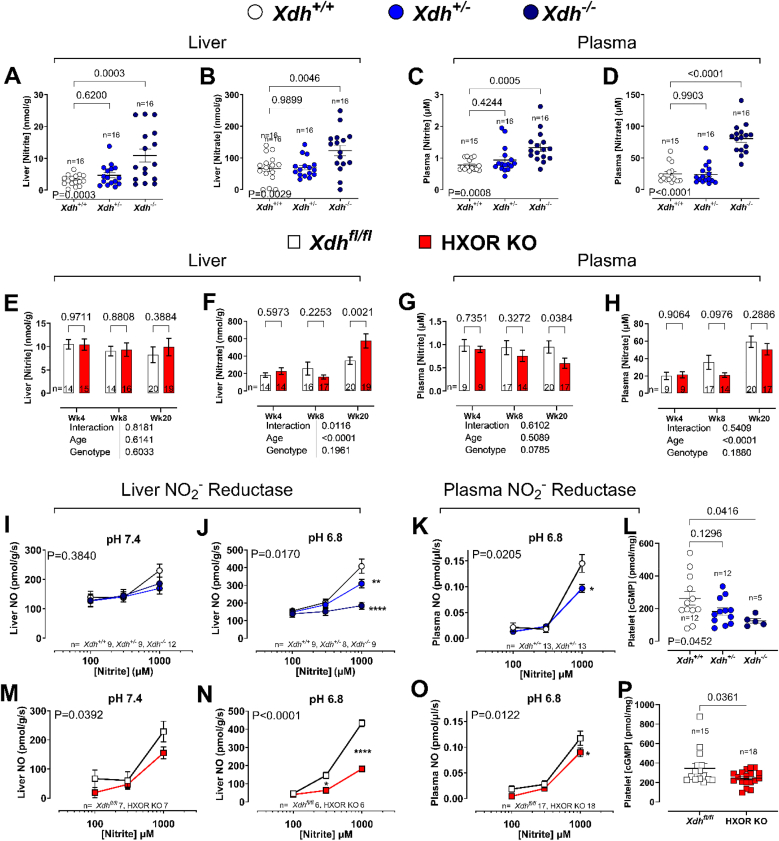
**XOR functions as a nitrite reductase.** Levels of liver **(A)** nitrite and **(B)** nitrate, and plasma **(C)** nitrite and **(D)** nitrate in 4-week-old global *Xdh* deleted mice and their wild type littermate controls. Levels of liver **(E)** nitrite and **(F)** nitrate, and plasma **(G)** nitrite and **(H)** nitrate in HXOR KO mice versus the *Xdh*^*fl/fl*^ wild type littermate controls. Nitrite reductase activity of liver homogenates at **(I)** pH 7.4, and **(J)** pH 6.8 and **(K)** plasma at pH 6.8 and **(L)** cGMP concentration of platelets isolated from blood samples collected from 4-week-old global *Xdh* deleted mice versus their wild type littermate controls. Nitrite reductase activity in liver homogenates, at **(M)** pH 7.4, and **(N)** pH 6.8 and **(O)** plasma at pH 6.8 **(P)** and cGMP concentration of platelets isolated from blood samples, collected from 20-week-old HXOR KO mice versus the *Xdh*^*fl/fl*^ wild type littermate controls. Data are shown as mean ± SEM of n mice (shown on individual graphs). Statistical significance was determined using one-way ANOVA followed by Sidak's multiple post-tests highlighting significant difference from *Xdh*^+/+^**(A-D and L),** using mixed effect analysis **(E**–**H),** two-way ANOVA **(M**–**O)** followed by Sidak's multiple *post hoc* tests shown as ∗P < 0.05, ∗∗P < 0.01, ∗∗∗P < 0.001 vs *Xdh*^*+/+*^ or *Xdh*^*fl/fl*^ at respective concentration, or unpaired Student's t-test **(L and P)**.

To explore whether *Xdh* deletion impacts circulating nitrite and nitrate levels, we measured both anions in tissues and blood of mice of ∼4 weeks of age to enable comparisons between *Xdh*^*+/+*^, *Xdh^+/−^* and *Xdh*^*−/−*^ littermate mice. An allele-dependent elevation in liver and plasma nitrite and nitrate levels was observed in globally deficient mice (Fig. 2A–D). These changes were associated with an allele-dependent decrease in nitrite reductase activity at pH 7.4 and 6.8 in liver homogenates and plasma (Fig. 2I–K). Interestingly, in comparison to *Xdh*^*fl/fl*^ mice, HXOR KO mice expressed an increase in liver nitrate levels at 20 weeks (Fig. 2F) with a decrease in plasma nitrite levels (Fig. 2G). The age-dependent changes in anion levels, in the HXOR KO mice, were also associated with reduced nitrite-reductase activity in both liver and plasma in 20-week-old mice (Fig. 2M). To determine whether the reductions in nitrite reductase activity led to deficiencies in vascular NO, we assessed platelet cGMP levels as a marker of NO activity. Indeed, in both the *Xdh*^*−/−*^ and HXOR KO mice platelet cGMP was reduced in comparison to control littermate mice, *Xdh*^*+/+*^ and *Xdh*^*fl/fl*^ mice respectively, although this did not reach statistical significance for HXOR KO mice (Fig. 2L and P).

There is some suggestion that in the absence of functional XOR, compensatory changes may occur in eNOS expression and/or activity. To assess whether changes in eNOS might account for the above noted effects we assessed total eNOS mRNA (Fig. S2A and S2H) and protein expression (Figs. S2D, S2G, S2K and S2N), as well as phospho-eNOS expression (Figs. S2C, S2F, S2K and S2N) as a measure of activity, in liver and aorta homogenates. No differences were evident in the *Xdh* ^^+/−^^ and HXOR KO mice versus the control littermate *Xdh*^*+/+*^ and *Xdh*^*fl/fl*^ mice respectively. In the 4-week-old global homozygous deleted mice some increase in eNOS expression was evident (Fig. S2G) – although the ratio of phospho-eNOS to total expression was unaltered (Fig. S2F).

### 3.3 Hepatocyte *Xdh* deletion elevates BP and promotes cardiac remodelling

**Fig. 3:**
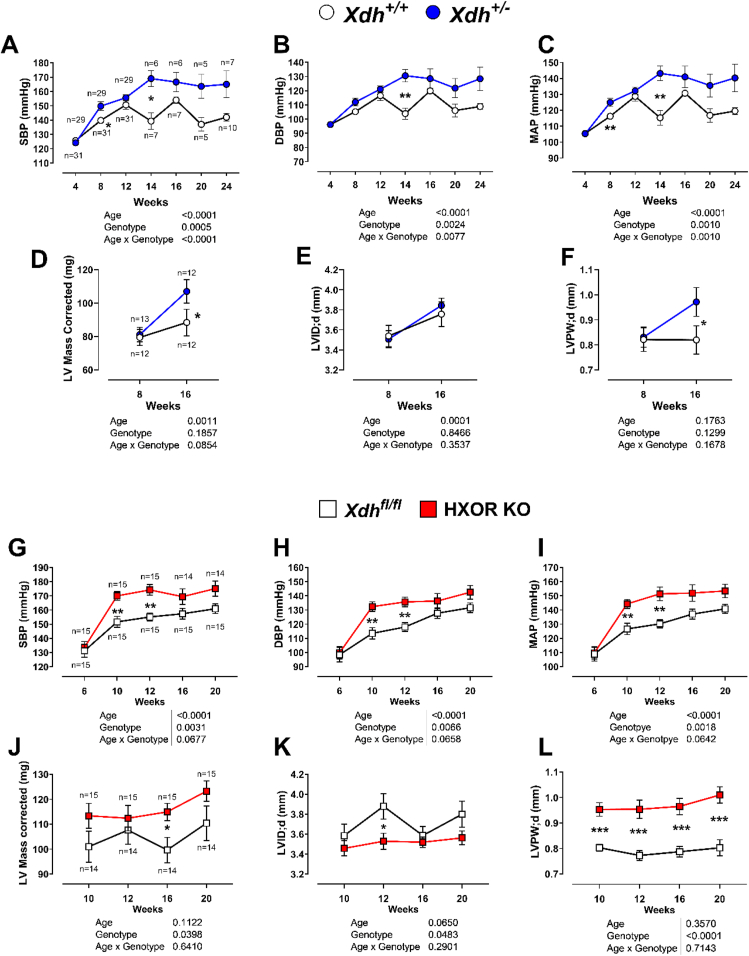
***Xdh*^*+/−*^ and HXOR KO mice exhibit raised blood pressure and left ventricular remodelling.**(A) Systolic, **(B)** diastolic, and **(C)** mean arterial pressure were raised in *Xdh*^*+/−*^ versus *Xdh*^*+/+*^ mice. Echocardiography data of *Xdh*^*+/−*^ versus *Xdh*^*+/+*^ littermates (8–16 weeks) showing **(D)** Short axis M-mode (SAX) LV corrected mass, **(E)** SAX LV internal diameter in diastole, and **(F)** SAX LV posterior wall thickness in diastole. **(G)** Systolic, **(H)** diastolic, and **(I)** mean arterial pressure were raised in HXOR KO versus *Xdh*^*fl/fl*^ mice. Echocardiography data of HXOR KO versus *Xdh*^*fl/fl*^ mice littermates (10–20 weeks) showing **(J)** SAX LV corrected mass, **(K)** SAX LV internal diameter in diastole, and **(L)** SAX LV posterior wall thickness in diastole. Data are shown as mean ± SEM of n mice (indicated in graphs **(A), (D), (G)** and **(H)** for the respective row. Statistical significance was determined using mixed-effect analysis followed by Sidak's multiple *post hoc* tests shown as ∗P < 0.05, ∗∗P < 0.01, ∗∗∗P < 0.001.

Since the non-canonical pathway for NO delivery has been shown to profoundly influence BP, this was measured in both mouse models over 24 weeks. At 4 weeks of age *Xdh*^*+/+*^ and *Xdh^+/−^* mice exhibited comparable BP. However, in agreement with previous findings [31] between 8 and 24 weeks of age *Xdh^+/−^* mice exhibited elevated systolic BP (SBP), diastolic BP (DBP) and mean arterial pressure (MAP) versus litter and age-matched *Xdh*^*+/+*^ mice (Fig. 3A–C). Importantly, this phenotype was replicated in mice with hepatocyte selective deletion of *Xdh,* with HXOR KO mice expressing elevated SBP, DBP and MAP from 10 weeks of age (Fig. 3G–I), implicating liver XOR in the BP effects observed in both mouse models.

Echocardiography revealed that *Xdh^+/−^* mice develop significant LV hypertrophy (Fig. 3D), at 16 weeks of age, following the onset of hypertension. The LV diastolic volume (Fig. S4K) and area (Fig. S4E) were significantly enlarged however; the diastolic internal diameter of the LV (Fig. 3E) was not affected. This LV hypertrophy was accompanied by significant thickening of the LV posterior wall (Fig. 3F) in *Xdh^+/−^* mice. No significant changes in LV systolic parameters were observed in *Xdh^+/−^* mice in either the short axis (Fig. S3) or parasternal long axis (Fig. S4). This compensatory LV diastolic wall thickening in *Xdh* ^**^+/−^**^ mice likely prevents a reduction in ejection fraction (Fig. S4C) and stroke volume (Fig. S3H), resulting in *Xdh^+/−^* mice having a cardiac output (Fig. S3J) comparable to that of *Xdh*^*+/+*^ littermates.

Similarly, HXOR KO mice displayed significant LV hypertrophy coinciding with the onset of hypertension (Fig. 3J). However, in contrast to the global KO model, the remodelling in HXOR KO mice appeared to be less compensatory and more maladaptive. HXOR KO mice displayed a significant reduction in LV volume (Fig. S6I) and area (Fig. S6F) in both diastole and systole (Figure S6G-H and Figure S6J-K),. This smaller LV area is attributed to a significant reduction in the diastolic internal diameter (Fig. 3K) and the reduced internal diameter in HXOR KO mice may be due to significant thickening of both the posterior (Fig. 3L and SG) and anterior walls (Fig. S5E and S5F). The pathological LV remodelling and reduced internal diameter in HXOR KO mice did not affect heart rate (Fig. S5L) or ejection fraction (Fig. S6C) but did significantly attenuate LV stroke volume (Figure S5K and), which in HXOR KO mice corresponds with a decreased cardiac output (Figs. S5J and S6E). To further assess LV mass, excised LVs were collected and weighed from 20-week-old HXOR KO and *Xdh*^*fl/fl*^ littermate mice. In accordance with the echocardiographic data, the LV mass normalised to the tibia length of HXOR KO mice is significantly greater than the wild type littermate controls (Fig. S5H).

Further assessment of diastolic function in HXOR KO mice revealed no single parameter showing a statistically significant change. However, a trending increase in the E/E’ ratio (Fig. S8H), LV myocardial performance index (MPI) using isovolumetric parameters (IV) (Fig. S8I), and LV MPI using non-filling time (NFT) (Fig. S8J) were evident. The impaired cardiac output in HXOR KO mice also corresponds with a trend towards reduced aortic velocity time integral (VTI) (Fig. S9A) relative to their littermate controls.

**Fig. 4:**
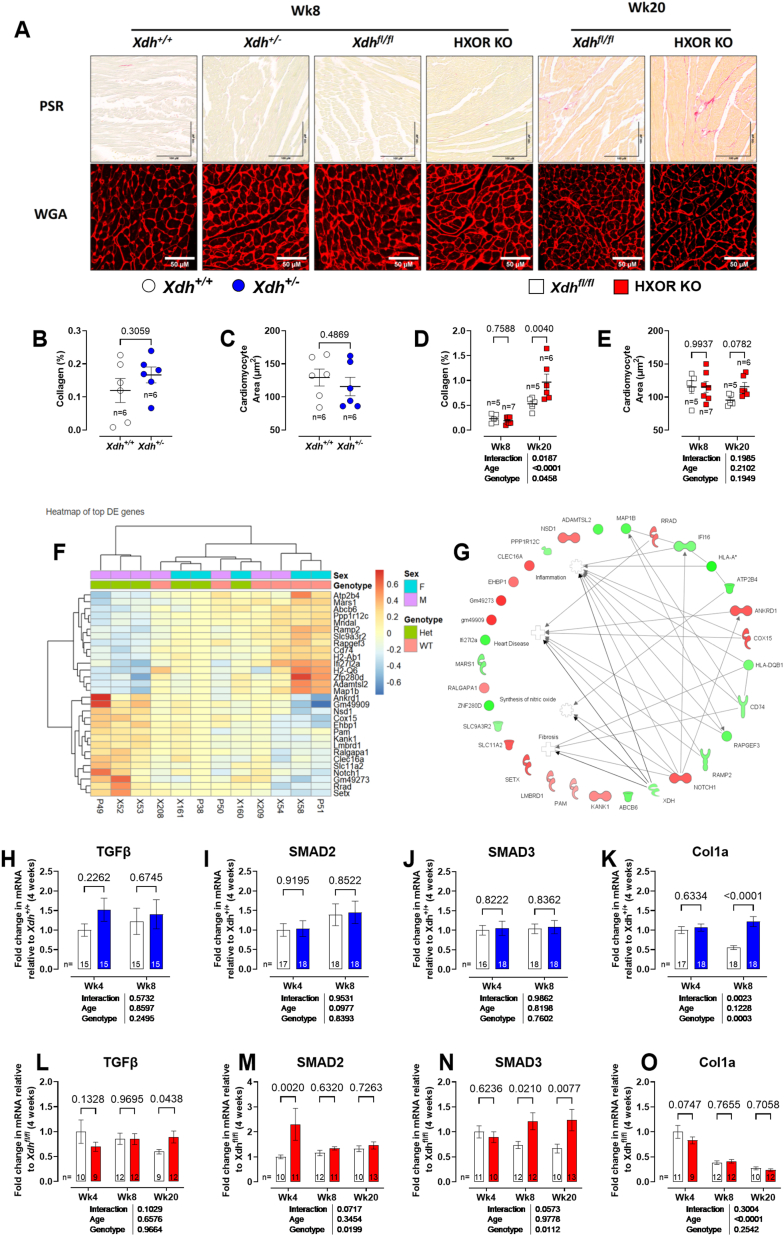
**Upregulation of profibrotic pathways in *Xdh*^*+/−*^ and HXOR KO mice. (A)** Representative images of picrosirius red (PSR) and wheat germ agglutinin staining (WGA) in sections of formalin fixed left ventricle (LV) (Scale bars are 100 μm and 50 μm respectively). Quantification of levels of **(B, D)** cardiac collagen deposition and **(C, E)** cardiomyocyte area in both the global *Xdh*^*+/−*^ and HXOR KO mice versus littermate controls. **(F)** Heatmap summary of all differentially expressed (DE) genes (q < 0.1) identified in total mRNA extracted from whole hearts of 8-week-old *Xdh*^*+/+*^ and *Xdh*^*+/−*^ mice. **(G)** Network plot demonstrating key disease and biological function annotations across DE genes. Longitudinal mRNA expression profiling in total mRNA extracted from whole hearts of *Xdh*^*+/−*^ and HXOR KO mice versus littermate controls investigating markers of fibrosis: **(H, L)** TGFβ, **(I, M)** SMAD2, **(J, N)** SMAD3, and **(K, O)** Col1a. Data are shown as mean ± SEM of n mice (shown on individual graphs). Statistical significance was determined using mixed effects analysis followed by Sidak's multiple *post*-*hoc* tests. **(D-E, H–O)** or unpaired Student's t-test **(B, C).** Uneven n values relate to sample loss due to technical failure or Routs exclusion.

Since *in vivo* functional studies suggested BP-induced changes in cardiac function occurred as early as 8 weeks of age and increased thereafter, we measured immunohistochemical markers of hypertrophy and/or fibrosis at both 8 and 20 weeks of age. At 8-weeks of age *Xdh*^*+/+*^ and *Xdh^+/−^* mice expressed comparable collagen content and cardiomyocyte area (Fig. 4B and C), and a similar pattern was also observed in 8-week-old HXOR KO mice vs *Xdh*^*fl/fl*^ (Fig. 4D and E). However, in 20-week-old HXOR KO, mice when both raised BP and cardiac dysfunction are strongly expressed, elevated collagen deposition was observed within the heart (Fig. 4D) associated with a trend towards increased cardiomyocyte area (Fig. 4E).

To explore the potential signalling pathways that might drive these effects we conducted bulk RNA sequencing of cardiac homogenates of 4-week-old mice to better explore the initiating signalling pathways triggered by an absence of *Xdh.* Our analyses demonstrate an upregulation of pro-fibrotic pathways in the young *Xdh* ^*+/−*^ mice, indicative of the triggering of cardiac remodelling compared to their *Xdh*^*+/+*^ littermate controls (Fig. 4F and G). Accordingly, qPCR of key cardiac pro-fibrotic signalling pathways at 4–20 weeks suggested some (albeit modest) activation of the TGFβ−SMAD-Col1a pathway with a trend to increase in TGFβ expression in the 4-week-old *Xdh^+/−^* mice and increased Col1a at 8 weeks (Fig. 4H–K). In the HXOR KO mice relative to their *Xdh*^*fl/fl*^ littermates SMAD2-3 was upregulated (Fig. 4L–O). These findings support the view that the pro-fibrotic effects in the heart were likely due to an absence of circulating XOR. Collectively these data add further support to the view that impaired liver XOR-dependent nitrite reductase activity in HXOR KO mice results in a phenotype resembling that of early heart failure with a preserved ejection fraction (HFpEF).

### 3.4 Reduced hepatic nitrite reductase activity leads to systemic inflammation and endothelial dysfunction

Interestingly, bulk RNA seq analyses of whole heart homogenates also highlighted the upregulation of pro-inflammatory signalling pathways in *Xdh^+/−^*mice (Fig. 4G). To explore this further we conducted qPCR of cardiac supernatants in both the *Xdh^+/−^* and HXOR KO mice relative to their littermate controls for a range of proinflammatory mediators (Figs. S12 and S13). These results reveal mild upregulation of pro-inflammatory mediators in the hearts of ageing HXOR KO mice, particularly TNFα (Figs. S12G and S13G).

**Fig. 5:**
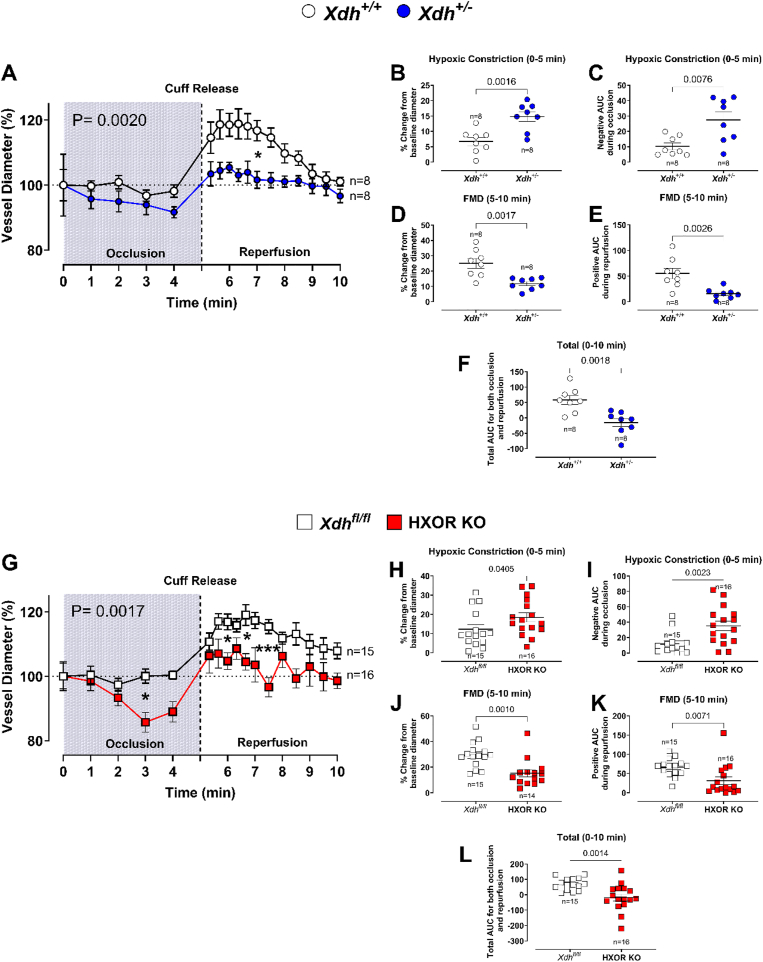
**HXOR KO and *Xdh***^*+/−*^**mice have impaired flow mediated dilatation (FMD).** FMD was assessed in both 20-week-old **(A)***Xdh^+/−^* and (**G)** HXOR KO mice versus littermate controls. An occlusion cuff placed above the left hind leg knee was inflated to 300 mmHg at T = 0 min to occlude blood flow through the left arteria iliaca externa. Time frame of occlusion is represented by the blue box. At T = 5 min the cuff was released (represented by the vertical dotted line). **(B and H)** show the maximal constriction of the vessel recorded during the occlusion phase expressed as a percentage change from the baseline diameter. (**C and I**) The negative area under the curve (AUC) for vessel constriction during the 5-min occlusion phase is shown. The maximal FMD recorded post cuff release **(D and J)**. The positive AUC for vessel dilation during the 5-min reperfusion phase is shown in **E and K**. The total net AUC for the vessels during the 10-min recording (positive AUC – negative AUC) is shown in **F and L**. Data are shown as mean ± SEM of n mice (shown on individual graphs). Statistical significance was determined using two-way ANOVA followed by Sidak's multiple *post-hoc* tests **(A, G)** with **p***ost hoc* test at an individual timepoint shown as ∗P < 0.05 or using unpaired Student's t-test **(B–F and H-L)**.

In cardiovascular disease, systemic inflammation drives endothelial dysfunction that often precedes cardiac dysfunction. Endothelial NO deficiency plays a critical role in this process. Therefore, we assessed endothelial function *in vivo*. As expected, cuff inflation to occlude the external iliac artery followed by cuff release, led to a prominent increase in vessel diameter due to shear stress-induced endothelial activation in control *Xdh*^*+/+*^ mice (Fig. 5A). Interestingly, whilst there was minimal change in the vessel diameter during the occlusion period (1–5 min) in *Xdh*^*+/+*^ wild type mice, in *Xdh^+/−^* mice there was a significant decrease in vessel diameter (Fig. 5B and C). Additionally, after cuff release, *Xdh^+/−^* mice demonstrated significantly reduced vessel dilation compared to *Xdh*^*+/+*^ mice (Fig. 5D and E). In HXOR KO mice a similar response profile to both occlusion (vasoconstriction) and shear stress (reduced FMD) relative to their littermate controls was observed (Fig. 5D–F). These findings suggest that hepatocyte XOR plays a crucial role in maintaining systemic endothelial function and blood flow.

### 3.5 XOR-deficiency triggers hepatic inflammation driving systemic inflammation and endothelial dysfunction

**Fig. 6:**
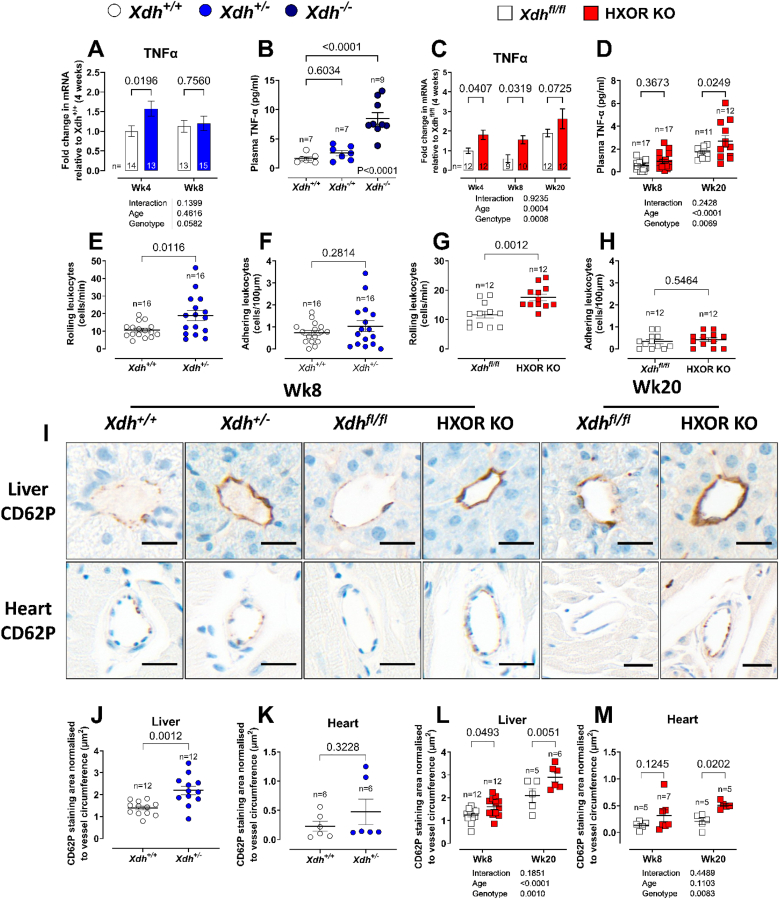
***Xdh*^*+/−*^ and HXOR KO mice exhibit elevated systemic inflammatory state.** Longitudinal TNFα mRNA expression in liver homogenate and plasma TNFα levels in 4-week-old of *Xdh*^*+/−*^ mice versus *Xdh*^*+/+*^ littermates (**A-B)** and HXOR KO mice versus *Xdh*^*fl/fl*^ littermates **(C)** and **(D).** Brightfield intravital microscopy was used to determine the number of **(E and G)** rolling and **(F and H)** adhering leukocytes in mesentery vessels of 4-5-week-old *Xdh*^*+/−*^ and HXOR versus wild type littermate mice. Representative images of CD62P staining (**I**) in vessels of liver and heart and their quantification **(J and L)** and **(K and M)** of *Xdh*^*+/−*^ and HXOR versus wild type littermate mice. Data are shown as mean ± SEM of n mice (shown on individual graphs). Statistical significance was determined using two-way ANOVA followed by Sidak's multiple *post-hoc* tests **(A, C, L, and M)** or using unpaired Student's t-test **(B, D, F–K).** Uneven n values relate to technical failure or exclusion using ROUTS. Uneven n values relate to technical failure or exclusion using ROUTS.

Since the mild inflammatory effects were observed in both the liver-specific knockout and the globally deleted mice, we hypothesized that the cardiac dysfunction and mild inflammation in the heart might be secondary to a systemic inflammation that originated within the liver of these animals. qPCR analyses of key inflammatory mediators revealed increased expression of TNFα mRNA, particularly at young ages, in the liver of both *Xdh*^*+/−*^ (Fig. 6A) and HXOR KO mice relative to their wild type littermates (Fig. 6C). This observation was confirmed by measurement of TNFα protein expression in the plasma of 4-week-old *Xdh*^*−/−*^ mice (Fig. 6B) and a trend to increase in 20-week-old HXOR KO mice (Fig. 6D) versus their respective wild type littermate controls. No statistically significant alterations were observed in other surveyed inflammatory mediators, except for NLRP-1 and TLR5 in HXOR KO mice (Figs. S14, S15, and S16).

That the systemic inflammatory state with associated dysfunctional endothelium led to important functional detrimental consequences was obtained from intra-vital microscopy studies. In 4–5-week-old mice, leukocyte rolling was enhanced in *Xdh^+/−^* compared to *Xdh*^*+/+*^ mice (Fig. 6E), while leukocyte adhesion remained comparable between the groups (Fig. 6F). Similarly, HXOR KO mice exhibited increased leukocyte rolling (Fig. 6G) albeit no change in adhesion (Fig. 6H) relative to the *Xdh*^*fl/fl*^ littermates.

Interestingly, there were no observed differences in the number of CD45^+^ cells within the liver or heart of 8 or 20-week-old *Xdh* ^*+/−*^ or HXOR KO mice relative to their littermate wild type controls (Figs. S17 and S18). However, there was an increase in the expression of the NO-sensitive adhesion molecule CD62P (P-selectin) in endothelial cells of both the liver and heart of *Xdh^+/−^* and HXOR KO mice (Fig. 6J–M). No difference in ICAM or VCAM-1 mRNA expression by genotype was observed (Fig. S17J) in the livers of either mouse model except an apparent post-hoc reduction at 20 weeks in HXOR versus the *Xdh*^*fl/fl*^. These findings are broadly consistent with a phenotype of enhanced leukocyte rolling without progression to transendothelial migration. That the enhanced leukocyte rolling is due primarily to changes in endothelial activity rather than direct changes in leukocyte activity is supported by the lack of difference in circulating leukocyte number (Table S4) or activation state (Table S5) between *Xdh^+/−^* and *Xdh*^*+/+*^ mice. These findings suggest the pivotal role of hepatocyte-derived XOR in triggering hepatic inflammation that initiates a systemic pro-inflammatory response leading to generalised endothelial dysfunction, elevated BP, and ultimately cardiac remodelling.

### 3.6 *Xdh* deletion prevents dietary nitrate-induced protection against ischaemia-reperfusion injury

**Fig. 7:**
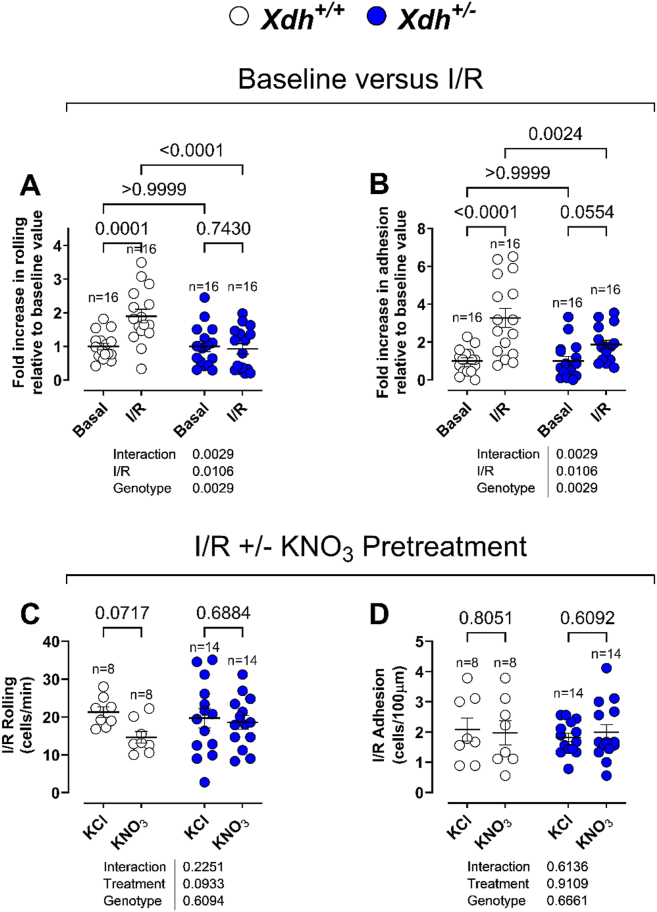
**XOR enhances leukocyte recruitment under I/R injury, which is reduced with inorganic nitrate.** Brightfield intravital microscopy was used to visualise leukocyte **(A)** rolling and **(B)** in mesentery vessels following 30 min of localised ischaemia and 45 min of reperfusion in 4-5-week-old *Xdh*^*+/−*^ versus wild type littermate mice. At 3–4 weeks of age drinking water was supplemented with either 15 mM KNO_3_ (nitrate intervention) or 15 mM KCl (vehicle) for 1 week prior to I/R injury. At the end of the 1-week KNO_3_/KCl pretreatment I/R injury was induced, as previously stated and leukocyte **(C)** rolling and **(D)** adhesion was assessed. Data are shown as mean ± SEM of n mice (shown on individual graphs). Statistical significance was determined using Two-way ANOVA followed by Sidak's multiple *post-hoc* tests.

We next investigated the impact of XOR deletion on ischaemia/reperfusion (I/R) injury-induced leukocyte activation and recruitment, given the previous implication of XOR in this response. As expected *Xdh*^*+/+*^ mice exhibited elevated levels of basal leukocyte rolling: absolute basal cell rolling in *Xdh*^*+/+*^ mice was 10.6 ± 1.0 and in *Xdh^+/−^* mice was elevated at 18.8 ± 2.9 cells/min (P < 0.011). For cell adhesion these values were 0.7 ± 0.1 cells for *Xdh*^*+/+*^ and 1.0 ± 0.2 cells for *Xdh* ^*+/−*^ mice (P = 0.28). In response to I/R injury compared to basal, leukocyte rolling (Fig. 7A) and adhesion were enhanced (Fig. 7B). However, these effects were absent in *Xdh^+/−^* mice, with only a small elevation in leukocyte adhesion observed under I/R injury conditions (Fig. 7A and B). These findings suggest that while XOR is protective under physiological conditions, it contributes to leukocyte recruitment during I/R injury. In addition, in *Xdh*^*+/+*^ mice, treatment with inorganic nitrate intervention resulted in a trend to reduction in I/R-induced leukocyte rolling (Fig. 7C) with no change in adhesion (Fig. 7D). In contrast this intervention had no effect in *Xdh^+/−^* mice. Analysis of circulating leukocyte numbers in mice supplemented with inorganic nitrate showed a statistically significant reduction in neutrophil and inflammatory monocyte counts in *Xdh*^*+/+*^ mice, which was attenuated in *Xdh^+/−^* mice, with no changes observed in other leukocyte subtypes (Table S6). To assess if this was due to neutrophil mobilisation, we assessed CXCR2 expression on circulating and bone marrow neutrophils and inflammatory monocytes but found no significant changes with inorganic nitrate treatment (Table S7). Moreover, the expression of CD162, CD62L and CD11b on various leukocyte populations remained unaltered with inorganic nitrate intervention (Table S8). Collectively, these data suggest that XOR plays a functional role in mediating leukocyte recruitment under physiological conditions and may also help attenuate I/R-induced leukocyte recruitment in the presence of increased circulating nitrite.

Given the observed modulation in leukocyte recruitment, both at baseline and following nitrate intervention, we also investigated whether circulating leukocytes express XOR. We did not detect extracellular XOR expression on any leukocyte subtype in *Xdh*^*+/+*^ or *Xdh^+/−^* mice (Table S9). In contrast, intracellular XOR expression was detected in all leukocyte subtypes assessed, albeit with varying degrees of expression (Table S9). In *Xdh*^*+/+*^ mice, neutrophils exhibited the greatest level of XOR expression, followed by monocytes, lymphocytes and B cells. In *Xdh^+/−^* mice, a similar expression profile was observed, albeit the level of XOR expression in neutrophils was significantly reduced. These data suggest that leukocytes, in particular neutrophils, have the capacity to synthesise XOR, implying their ability to reduce nitrite to NO.

## 4 Discussion and conclusion

Herein, we demonstrate a critical role for hepatic XOR mediated bioconversion of nitrite to NO in maintaining vascular and cardiac health. This XOR-derived NO suppresses leukocyte activation, maintains healthy BP and cardiac function as well as preventing leukocyte activation in pathological scenarios such as I/R injury. Collectively these findings implicate liver-derived XOR in sustaining cardiovascular physiology, challenging the view that the function of XOR in the cardiovascular system is solely one of driving disease pathology [[32], [33], [34]]. Moreover, these findings highlight the potential in targeting hepatic XOR through dietary nitrate delivery to prevent vascular damage by attenuating the systemic inflammation that drives vascular dysfunction and cardiovascular disease.

Using the global *Xdh* deletion mouse model [21] we confirmed previous observations indicating that reduced expression of XOR leads to reductions in the products of its activity i.e. UA and O_2_^−^/H_2_O_2_. However, in addition we show, for the first time, that this expected reduction in redox activity is accompanied by a clear allele-dependent reduction in nitrite reductase activity in tissues and blood, also reflected by alterations in nitrite and nitrate levels. We acknowledge that to visualise the differences very high nitrite concentrations were required in this ex vivo assay and that the NO production rates in plasma were low relative to what one might expect for functional activity. Further work should explore the physiological relevance of this NO production using alternative assays. Using the pterin fluorometric assay, we observed a greater proportion of XOR in the XDH form, vs XO, in the liver, whilst in plasma the proportions were comparable. This difference has been attributed previously to sulfhydryl oxidation once released into the circulation [35,36]. Since we identified nitrite reductase activity in both the liver and plasma, this observation suggests that both isoforms likely contribute to physiological levels of NO, despite some suggestions that this is not the case [37].

To further explore the role of XOR-derived NO in cardiovascular function we created a floxed mouse, using a CRISPR-Cas9 approach, to overcome the difficulties with survival associated with global deletion [30]. We focussed specifically upon deletion of hepatocyte *Xdh* since the liver is a primary source of circulating XOR implicated in driving the pathogenesis of cardiovascular disease [32,36,38]. This mouse is different from the recently published mouse model created by Kelley and co-workers using a Neo cassette approach [39]. Basic phenotyping of XOR expression and activity confirmed selective hepatic deletion and showed considerable similarity with the previously published mouse model. However, some important differences between the models were identified. Whilst in the previous study an approximate 50–60 % reduction in plasma XOR activity was evident [39], in this CRISPR-Cas9-induced deletion mouse this was not the case; a finding further supported by our observations that there was no significant change in plasma UA level. This discrepancy between the models may relate to the method used to quantify XOR activity, potential off-target deletion, or even technical issues regarding the blood collection method. In our study, blood was collected using citrate as an anticoagulant to avoid heparin-mediated scavenging of GAG-bound XOR found on RBCs or endothelial cells [20,40]. Our analysis shows that the total organ XOR protein expression was unaffected in other tissues collected from the HXOR KOs versus the littermate controls indicating hepatocyte selectivity. It is possible that, in addition to the liver, circulating XOR levels may arise from release by other high XOR-expressing organs, such as for instance the lung or adipose tissue, and thus compensating, at least in part, for the absence of liver-derived circulating XOR [41]. However, we show important functional effects of hepatocyte-specific XOR deletion upon the cardiovascular system, coupled with reduced nitrite reductase activity in organs distant from the liver. These results suggest that cell surface localisation, whilst small in comparison to the total tissue expression level, exerts local effects key to sustaining cardiovascular health.

In the HXOR KO mice, we identified alterations in NO_x_ levels with a rise particularly in nitrate levels in the liver. We suggest that the elevation in liver and plasma nitrite and nitrate, in both models, is due to reduced XOR-dependent conversion of nitrite to NO. We speculate that this leads to an accumulation of plasma nitrite due to less utilisation/metabolism. Nitrite has a short half-life, primarily due to its oxidation to nitrate, and the rise in nitrate levels likely reflects re-direction of nitrite, from nitrite reduction, down this route of metabolism [42,43]. We also speculate that the attenuation of plasma nitrite in the HXOR KO animals is associated with decreased systemic NO bioavailability and oxidation into nitrite since we observed clear reductions in the levels of platelet cGMP. It is noteworthy that in the *Xdh*^*−/−*^ animals renal function declines rapidly as they age due to the build-up of purine crystals in the kidney [21,30], and thus it is possible that disordered renal clearance contributes in some way to the alterations of NOx levels. However, since there is no evidence for renal dysfunction/failure in either the global heterozygotes or those with selective hepatocyte deletion, it is unlikely that failure of renal clearance underlies the changes seen. These findings support the suggestion that hepatocyte XOR is unlikely to be the exclusive source of circulating physiological XOR, and that XOR released from other organs into the circulation express nitrite reductase activity. These studies also support the many *in vitro* observations implicating XOR, using pharmacological inhibitors i.e. allopurinol and febuxostat, demonstrating nitrite reductase activity in tissues collected primarily from ‘cardiovascular disease’ scenarios in both experimental animals and patients (for review [15,16,44]). Herein, we now demonstrate that, in contrast to some recent suggestions [45], in addition to acting as a nitrite reductase in disease, that the nitrite reductase activity of XOR activity also plays an important role in cardiovascular physiology.

Due to the critical role of vascular NO in BP control, we reasoned that if nitrite-derived NO is important in maintaining physiology that XOR deletion, and the loss of nitrite-derived NO, would influence BP. Both HXOR KO and *Xdh^+/−^* mice displayed increasing BP with age versus their respective littermate controls, despite normal BP at 4–6 weeks of age. This is in accord with a previous study showing modest SBP elevation at 2 months in *Xdh^+/−^* mice, increasing further at 4 and 18 months [31]. Our findings suggest that to sustain healthy BP during ageing that there is an increased importance of XOR as mice move from the juvenile to the adult. Notably, previous work [31] also demonstrated *ex vivo* impaired endothelial-dependent relaxation of aortic rings of 4-month-old *Xdh^+/−^* mice. Consistent with these findings, we observed that elevated BP in *Xdh^+/−^* mice was associated with endothelial dysfunction measured *in vivo*, as indicated by an impaired FMD response. Importantly, the observed hypertension and endothelial dysfunction occurred in the absence of changes in circulating UA levels but was associated with reduced systemic XOR nitrite reductase activity and diminished NO bioavailability. Since reduced endothelium-derived NO bioavailability [46,47] is a known driver of hypertension, we propose that the hypertension observed in these genetic models is attributable to decreased liver-derived XOR-dependent NO generation at the endothelial cell surface. For these studies we used tail-cuff BP measurement rather than telemetry, employing gold-standard protocols recommended recently in updated guidelines (including sufficient sample size) [48]. The reasons for this choice relate to a need to measure BP as the mice age, with surgery for implanting and placing probes at 4 weeks through to 20 weeks of age impractical.

The mechanism by which XOR influences vascular tone and thus BP has long been debated but remains uncertain. Hyperuricemia has been associated with hypertension [49], however, in contrast, a recent study has suggested that plasma XOR activity, not serum UA, is positively associated with BP [50]. Additionally, whilst lower levels of oxidative stress have been associated with BP, this association was absent at higher oxidative stress levels [50]. There are also meta-analyses suggesting that XOR inhibition lowers BP [51]. But it should be noted that there are also studies showing no effect on BP [52,53]. Collectively, these observations highlight the current confusion regarding the role of XOR on BP [54,55]. We suggest that this complexity arises from a lack of consideration of the full spectrum of XOR activity. Our data demonstrates that a single allele deletion of *Xdh* results in an elevation of BP, contrasting with suggestions that pharmacological XOR inhibition results in a small but significant lowering of BP in certain patient cohorts [54,55]. A potential explanation for these conflicting data is that inhibition of XOR will result in a lowering of UA and ROS production but also a loss of XOR-mediated nitrite reduction. It has been suggested that in a scenario where conventional endothelial NO generation is prevented, i.e. the eNOS KO mouse, that there is a compensatory rise in XOR activity, to restore NO bioavailability [56]. This could lead to speculation that any apparent changes in levels of NO or its metabolites may be related to alterations in other sources of NO [57]. This possibility, however, is unlikely in the current setting since neither eNOS nor phospho-eNOS expression were altered in either genetic model. Why genetic deletion might expose its nitrite reductase activity and effect on BP and not pharmacological inhibition is uncertain. However, the impact of XOR inhibitors has been assessed in the setting of disease, generally hypertension or CAD, where endothelial and eNOS dysfunction is very prominent and circulating nitrite levels are thus low. In such a scenario XOR-dependent nitrite reductase activity will be limited due to low substrate levels. In the mouse models shown herein eNOS activity is intact and we speculate that this results in provision of nitrite as a substrate for XOR in physiology. Indeed, in wild type mice in this study we demonstrate plasma concentrations of ∼0.7–1.0 μM and 3–10 nmol/g tissue protein in liver. We suggest that this ‘storage’ form [58] of nitrite is utilised by XOR in the setting of the physiological hypoxia invoked by the cuff inflation, and that following *Xdh* deletion this reaction does not take place.

Our findings also underscore a critical role for XOR-dependent nitrite reductase activity in maintaining vascular tone under physiological hypoxia conditions, (i.e. during the brief occlusion phase required to trigger FMD), where eNOS-derived NO generation decreases due to its O_2_ dependence. This indicates that vascular XOR activity is modulated by shear stress and highlights its compensatory role in NO generation under reduced O_2_ tension [59,60]. Importantly, our results also demonstrate that this dependency is mediated by liver-derived XOR, likely bound to the endothelium. Recently, Cortese-Krott and co-workers, demonstrated that the FMD response of the iliac artery is completely absent in endothelial cell specific eNOS-ablated mice. However, these mice did not exhibit the enhanced vasoconstriction observed in our models during occlusion [26]. This supports our speculation that XOR-dependent nitrite reductase activity is upregulated in hypoxic environments to compensate for impaired eNOS function. Indeed, eNOS and XOR appear to operate in tandem to maintain vascular NO delivery. When eNOS activity is compromised, compensatory XOR-dependent NO generation increases to preserve vascular homeostasis [56]. Our findings extend this understanding by highlighting the pivotal role of XOR-dependent nitrite reductase activity in mitigating hypoxia-induced vasoconstriction and preserving endothelial function under conditions of reduced oxygen tension, in conjunction with the normal response of the endothelial to shear stress/blood flow. More recently there have been observations demonstrating sulfide/polysulfide-induced XOR-dependent reduction of NO_2_^−^ to NO [61], whether the nitrite reductase activity evidenced in this study involves such a pathway is unknown and warrants further investigation.

Prolonged elevated BP impacts cardiac function, and indeed, in the global deletion mice, raised BP precedes the cardiac phenotype. Interestingly, in HXOR KO mice a reduction in cardiac output alongside increased left ventricular posterior wall (LVPW) thickening and decreased left ventricular internal diameter (LVID) was observed. The differences in cardiac phenotypes—compensatory LV hypertrophy in the global heterozygous KO model, versus maladaptive remodelling in the homozygous hepatocyte KO model, likely highlights the distinct role of hepatocyte-derived XOR in cardiovascular homeostasis. The HXOR KO mice developed maladaptive cardiac –remodelling early and by 6 weeks of age, before the apparent onset of hypertension, indicating a potential BP-independent component. Indeed, our previous studies [23] have identified both BP-dependent and independent pathways underlying beneficial effects of activating the non-canonical pathway, through dietary nitrate delivery, in the setting of a severe cardiac dysfunction phenotype. Both pathways are dependent upon NO activity, but the latter is mediated by NO-driven suppression of the TGFβ-SMAD-Col1a pathway of fibrosis. Indeed, this latter possibility likely explains, at least a component of the effects, since upregulated mRNA expression for both TGFβ and Col1a were evident in the hearts of both *Xdh*-deficient models. Importantly, the cardiac changes, in both models, are consistent with a heart failure phenotype with diastolic dysfunction. These observations are in contradiction to a recent study using the Neo cassette approach to hepatic XOR deletion, where no differences in LV structure or function in comparison to *Xdh*^*fl/fl*^ mice were observed [39]. The reason for this discrepancy may relate to the use of a mixed-age population of mice, power of the study and to the sex utilised in the Kelley study. In this study mice were 6–13 weeks old, with only n = 7 per group and all male mice were used. Our study used equal numbers of male and female mice and aged-matched littermates, as well as assessment of a time-course exposing important age-dependent changes [62].

Mechanistically, our findings link the XOR-dependent vascular dysfunction temporally with inflammation. Within the liver, heart and plasma we saw consistent elevations in a key early cytokine signal in the inflammatory response i.e. TNFα. These differences overall appeared to increase with age, particularly in the HXOR KO, and followed a temporal pattern with rises in the cytokine in the liver at younger ages, followed by appearance with age in the plasma and then the heart. We suggest that the removal of *Xdh* from the liver eliminates an NO-mediated brake-mechanism leading to a proinflammatory phenotype within the liver and release of TNFα into the systemic circulation causing the consequent endothelial dysfunction and cardiac phenotype observed.

We also observed an increased albumin-bilirubin (ALBI) score, indicative of liver dysfunction. Impaired liver function affects the synthesis and metabolism of vasoactive substances, disrupts bile acid regulation, and contributes to the dysregulation of systemic hemodynamics. An increased ALBI score is associated with increased mortality risk in heart failure patients [63]. Whilst HXOR KO mice did not express increased levels of traditional markers of hepatocyte damage (ALT, AST, and ALP) this could be attributed to the collection of plasma used for the biochemical analysis interfering with the sensitivity of the assays [64]. However, ALBI score has demonstrated an ability to show earlier indications of liver disease than the traditional prognostic biomarkers [65].

Since a key property of endothelium-derived NO is its anti-inflammatory effect [66] we used intra-vital microscopy to assess the impact of XOR deletion on this parameter. Under basal conditions leukocyte rolling and adhesion were elevated in *Xdh^+/−^* mice compared to *Xdh*^*+/+;*^ a similar effect was observed in HXOR KO mice vs *Xdh*^*fl/fl*^. This contrasts with a study in cats treated with allopurinol (under basal conditions) where no change in leukocyte adhesion or emigration was evidenced compared to control [67]. A potential explanation for this discrepancy is that the cats were administered heparin before assessment. Heparin will compete with endothelial GAGs, to which circulating XOR binds, and thus reduce surface endothelium XOR expression [68]. We suggest that there is a dual action of endothelial GAG-bound XOR: purine degradation resulting in superoxide and hydrogen peroxide production both of which elicit leukocyte recruitment [69], but also nitrite reduction generating NO that inhibits leukocyte recruitment [70,71]. Our observation suggests that under basal conditions, the reduction in NO production has a more prominent effect on leukocyte recruitment than the reduction in ROS generation.

It is well accepted that NO reduces leukocyte endothelium interactions and hence recruitment. The mechanisms by which NO has been proposed to do this is predominantly via suppression of CD62P, and also some evidence that NO interferes with ICAM-1, CD62E and VCAM-1 expression on endothelial cells [[72], [73], [74]]. Indeed, in *Xdh^+/−^* and HXOR KO mice an increase in endothelial CD62P expression was observed in line with previous data demonstrating that NO regulates endothelial CD62P expression [75], however no significant effects upon ICAM-1 or VCAM-1 expression were observed. These results suggest that under basal conditions, hepatic XOR likely binding to endothelium via interaction with GAGs leads to XOR-dependent nitrite derived NO that plays a role in sustaining a quiescent endothelium. Since XOR is implicated in I/R injury (for review see Ref. [76]); an effect attributed to XOR-derived ROS [69] and oxidative stress-induced expression of TNFα and MCP-1 [77], we assessed the impact of XOR deletion in a mesenteric artery I/R injury model. We observed an increase in leukocyte recruitment in *Xdh*^*+/+*^ mice subjected to I/R injury, with no change in *Xdh* ^*+/−*^ mice; an effect concordant with observations by others assessing the impact of renal I/R injury in a similar mouse [77]. Collectively our observations confirm that reducing XOR-derived ROS in the setting of I/R injury is beneficial. But that under physiological conditions XOR-derived NO is important for maintaining vessel homeostasis to limit leukocyte recruitment and vascular dysfunction. There are limited studies investigating XOR expression on leukocytes however we identified XOR expression in neutrophils (others have suggested XOR expression in macrophages predominantly [78,79]). However, we saw no change in activation state of any leukocyte subtype assessed, supporting our contention that the anti-leukocyte effects observed likely relate to the activity of endothelium-bound XOR.

A complication of XOR inhibition *in vivo* is that using allopurinol/febuxostat or genetic deletion, will inhibit UA, ROS and NO production making identification of which pathways are the key drivers for any phenotype complex. An alternative method for targeting XOR is by providing an excess of substrate that would drive one of the synthesis pathways. In our study, dietary inorganic nitrate treatment elevated circulating nitrite in wild type and XOR-deleted mice. This rise in nitrite was associated with a reduction in leukocyte rolling in *Xdh*^*+/+*^ mice subjected to I/R injury, whilst there was no effect in *Xdh*^*+/−*^, confirming XOR as a nitrite reductase and demonstrating a functional effect *in vivo*.

Given that both global *Xdh^+/−^* and HXOR KO mice exhibit increased BP, endothelial dysfunction, and cardiac remodelling, our data support the concept of the “repurposing” of XOR [15] to deliver therapeutic benefit, rather than its pharmacological inhibition. The phenotype observed in our XOR KO models may explain the increased all-cause and cardiovascular mortality effects of the XOR inhibitor febuxostat in the CARES trial [80]. Our findings suggest that the positive physiological role of XOR-dependent nitrite reductase activity, particularly in preserving NO bioavailability, may be compromised by XOR inhibition, potentially contributing to these adverse outcomes. It is important to note that rodents are thought to exhibit substantially higher XOR expression and activity compared to human. Comparisons in the mRNA transcript levels identified ∼100-fold lower in human tissues versus mouse liver, kidney, and lung [81]. Although, detail regarding the provenance of the human tissue was not provided and thus some caution interpreting these observations is apposite. However, it is known that due to an absence of uricase in humans, lost through evolution, lower XOR expression and/or activity makes sense. Lower levels at least in the oxidase activity of XOR has been demonstrated in humans versus rabbit, pig, rat and bovine, although importantly this does not appear to be the case for the dehydrogenase activity [82,83]. Such a species difference could accentuate the impact of XOR deletion in mouse models and should be considered when extrapolating these findings to human physiology. Nevertheless, this study highlights the need to critically reevaluate the use of XOR inhibitors in patients with cardiovascular disease.

In summary, we have demonstrated a critical role for the nitrite reductase activity of hepatic XOR *in vivo*. Our data support the hypothesis that as mice age hepatic XOR functions increasingly as a nitrite reductase elevating cGMP to sustain cardiovascular health under physiological conditions and in the apparent absence of hypoxia or acidosis. This latter point presents an equipoise that requires further investigation. Additionally, under pathophysiological conditions, associated with reductions in circulating nitrite and nitrate, XOR becomes a ROS generator contributing to disease pathology. We suggest that these findings highlight the potential for exploitation of XOR as a nitrite reductase, through nitrate/nitrite delivery, in sustaining cardiovascular health and in treating disease where XOR expression is elevated.

## Sources of funding

Nicki Dyson (FS/19/62/34901) and Lorna Gee (FS/13/58/30648) were funded by BHF (British Heart Foundation) MRes/PhD Studentships. The development of the hepatic mouse model was funded by The Barts Charity Seed Grant (MGU0380). Tipparat Parakaw was funded by an EU ITN (675111). Krishnaraj Rathod was funded by the National Institute for Health and Care Research (NIHR) (DRF-2014-07-008) and NIHR ACL. Gianmichele Massimo was funded by The Barts Charity Cardiovascular Programme (MRG00913). Claudia P Cabrera was supported by the National Institute for Health and Care Research Barts Biomedical Research Centre.

## CRediT authorship contribution statement

**Nicki Dyson:** Formal analysis, Investigation, Methodology, Writing – original draft. **Rayomand S. Khambata:** Data curation, Formal analysis, Investigation, Methodology, Writing – original draft. **Tipparat Parakaw:** Investigation, Writing – review & editing. **Gianmichele Massimo:** Investigation, Writing – review & editing. **Ngan HH. Khuat:** Investigation, Writing – review & editing. **Annika A. Noor:** Investigation, Writing – review & editing. **Lorna C. Gee:** Investigation, Writing – review & editing. **Ivy Lim:** Investigation, Writing – review & editing. **Umme Siddique:** Investigation, Writing – review & editing. **Andrew J. Sullivan:** Investigation, Writing – review & editing. **Jonathan W. Ho:** Investigation, Project administration. **Krishnaraj Rathod:** Investigation, Writing – review & editing. **Michael R. Barnes:** Formal analysis, Writing – review & editing. **Claudia P. Cabrera:** Formal analysis, Writing – review & editing. **Amrita Ahluwalia:** Conceptualization, Data curation, Formal analysis, Funding acquisition, Methodology, Project administration, Resources, Supervision, Visualization, Writing – original draft.

## Declaration of competing interest

The authors declare the following financial interests/personal relationships which may be considered as potential competing interests: Amrita Ahluwalia is a Co-Director of Heartbeet Ltd and IoNa Therapeutics seeking to identify therapeutic opportunities for dietary nitrate.

## Data Availability

Data will be made available on request.
